# Karydakis Flap Procedure in Patients with Sacrococcygeal Pilonidal Sinus Disease: Experience of a Single Centre in Istanbul

**DOI:** 10.1155/2013/807027

**Published:** 2013-05-14

**Authors:** M. Kamil Yildiz, Erkan Ozkan, Hacı Mehmet Odabaşı, Bülent Kaya, Cengiz Eriş, Hacı Hasan Abuoğlu, Emre Günay, Mehmet M. Fersahoglu, Süleyman Atalay

**Affiliations:** Department of 5th General Surgery, Istanbul Haydarpasa Numune Training and Research Hospital, 34668 Istanbul, Turkey

## Abstract

*Background*. The aim of this retrospective study was to evaluate the results of patients with sacrococcygeal pilonidal sinus who underwent surgery using the Karydakis technique. *Methods*. Two hundred fifty-seven patients with sacrococcygeal pilonidal sinus disease were treated by the Karydakis flap procedure between December 2003 and June 2011. Patients were evaluated with respect to age, gender, preoperative symptoms, duration of preoperative symptoms, history of pilonidal sinus surgery, early postoperative complications, recurrence rates, and cosmetic satisfaction. *Results*. There were 223 (86.8%) male and 34 (13.2%) female patients. The mean age of the patients was 27.15 ± 7.69 years. The most frequent symptom was seropurulent discharge (57.58%). Postoperative morbidity was noted in 24 patients (9.3%). The mean hospital length of stay was 3.34 ± 1.42 days. The cosmetic satisfaction rate was 91.06%. Recurrences were noted in 6 patients (2.3%). *Conclusion*. The Karydakis flap procedure is a safe treatment alternative for the surgical treatment of sacrococcygeal pilonidal sinus disease owing to the associated low complication rate, short hospital length of stay, rapid healing, and a high patient satisfaction rate.

## 1. Introduction

Pilonidal sinus disease is a chronic, recurrent disorder of the sacrococcygeal region which commonly occurs in young adults following puberty [1–3]. Pilonidal sinus disease constitutes a significant portion of the patients treated in surgery clinics worldwide [4]. In the etiopathogenesis of pilonidal sinus disease, it is commonly accepted that non-living hairs provoke a foreign body reaction subcutaneously, leading to abscess and sinus formation. While congenital factors were considered causal in the previous years, acquired factors are currently considered etiologic. Pilonidal sinus disease is common among young men and does not occur in childhood, which suggests an acquired etiology [5]. Complete removal of the sinus or sinuses and proper reconstruction are required to achieve full recovery and prevent recurrence. For this purpose, many surgical techniques and medical methods have been described. The aim of the present study was to evaluate the results of patients retrospectively who were operated on with the Karydakis flap technique.

## 2. Methods

Two hundred fifty-seven patients with sacrococcygeal pilonidal sinus disease treated with the Karydakis flap procedure between December 2003 and June 2011 at the 5th General Surgery Clinic of Istanbul Haydarpasa Numune Training and Research Hospital were evaluated with respect to age, gender, duration of preoperative symptoms, a history of pilonidal sinus surgery, early postoperative complications, recurrence rates, and cosmetic satisfaction. During the follow-up period, the patients were called for a follow-up visit by telephone. Patients' data were evaluated from their hospital records.

### 2.1. Surgical Procedure

The procedures were undertaken with the understanding and appropriate informed consent of each patient before surgery. The operations were performed under spinal or general anesthesia. The gluteal area, including the region surrounding the intergluteal sulcus, was shaved the day before surgery. Preoperatively, the patients were intravenously administered with 1 g of a 1st generation cephalosporin for prophylaxis. After positioning the patient in the prone position, the skin around the intergluteal sulcus was stretched using medical tape and the skin was cleaned using 10% povidone iodine. The sinus tract was removed down to the sacrococcygeal fascia by a semilateral elliptic incision, while keeping the sinuses of the pilonidal sinus disease at the centre of the elliptic incision. The central aspect of the semilateral elliptic incision was immobilized, a flap was placed on the contralateral side with cutaneous-subcutaneous fatty tissue, and a surgical drain was placed in the cavity for aspiration purposes after the medical tape stretching the skin was removed. The flap was advanced to the contralateral side, the tissue was fixed on the contralateral side crossing the sacrococcygeal fascia using number 1 Vicryl suture, and the natal cleft was transposed laterally. The skin was sutured using 3–0 polypropylene suture. An oral free-feeding regimen was initiated in the early postoperative period. The patients were advised to pay special attention to perineal hygiene following defecation. The aspiration drain was removed when the flow rate was <25 mL/day (in between 1–5 days). Oral antibiotics were continued until the end of the first postoperative week. Wound sutures were removed 10–12 days postoperatively. According to hospital records, the patients had been followed up weekly for the first 6 weeks, and then at 6-month intervals after discharge.

### 2.2. Statistical Analysis

Statistical analysis was performed using Statistical Program for Social Sciences (SPSS Inc., SPSS for Windows, Chicago, IL, USA, 1999) version 10. A *P* value <0.05 was considered statistically significant.

## 3. Results

The patient population consisted of 223 (86.8%) males and 34 (13.2%) females. The male-to-female ratio was 6.55. Pilonidal sinus disease was found to be significantly more common in males (*P* < 0.05). The mean age of the patients was 27.15 ± 7.71 years (range, 14–47 years). The mean age of male patients was 27.72 ± 7.81 years and the mean age of female patients was 23.44 ± 5.86 years. The most frequent symptom was seropurulent discharge (148 patients, 57.58%; Table 1). The mean duration of symptoms was 20.45 ± 32.56 days. Twelve patients (4.7%) had a history of pilonidal sinus surgery performed in other centres. Postoperative morbidity was noted in 24 patients (9.33%; Table 2). Patients who developed abscesses were treated by drainage, followed by leaving the wound open for secondary healing. Healing was achieved without the need for a second operation. The mean length of hospital stay was 3.34 ± 1.42 days. Recurrence of pilonidal sinus disease was noted in 6 patients (2.3%). The follow-up period was in between 1 and 9 years. The patients were asked whether or not they were satisfied with the appearance of the surgical scar, and the cosmetic satisfaction rate was found to be 91.06%. The average period for return to work was 15 days.

## 4. Discussion

The ideal technique for the treatment of sacrococcygeal pilonidal sinus disease is a controversial issue [6]. The main goal of treatment of the sacrococcygeal pilonidal sinus disease is the selection of the most appropriate technique that causes the least number of early postoperative complications, shortens the length of hospital stay, and results in the least number of long-term recurrences [7]. Numerous methods have been described as surgical treatment alternatives for sacrococcygeal pilonidal sinus disease, including primary oblique excision and closure, marsupialization, secondary healing, V-Y advancement flap, Z-plasty, Limberg flap, and Karydakis flap techniques [8, 9].

The Karydakis procedure is one of the most frequently used asymmetric flap techniques in the treatment of sacrococcygeal pilonidal sinus disease. This technique was described by Karydakis in 1973 and recurrence rates were reported to be <1% [10, 11]. Karydakis has suggested the following three factors responsible for the development of pilonidal sinus disease: hairs, the force that causes hair insertion, and formation of sinus in the skin [12]. Lateral transposition of the centrally located natal cleft by eliminating the above-mentioned factors is aimed in the Karydakis procedure, which effectively prevents recurrence [13].

Pilonidal sinus disease is common in the young, and especially the male population. The peak incidence for sacrococcygeal pilonidal sinus disease is encountered between 15 and 24 years of age. The symptoms of sacrococcygeal pilonidal sinus disease are rare before 15 years of age and after 40 years of age [13, 14]. Gurer et al. [15] reported that the mean age of patients treated using the Karydakis technique was 25.5 years (range, 18–41 years), and that 95% of the patients were males. Similarly, in their study, Bessa [16] reported that the mean age of the patients was 23.5 ± 4.3 years (range, 17–39 years) and that 92.7% were males. In the present study, the mean age of the patients with sacrococcygeal pilonidal sinus disease who underwent the Karydakis procedure was 27 ± 7.71 years (range, 14–57 years) and 86.8% of the patients were males.

Several postoperative morbidities causing delays in healing, loss of workforce, and an increase in the length of the hospital stay have been reported following treatment of pilonidal sinus disease, such as wound site infections, wound separation, abscess formation, and seroma formation. The Karydakis technique provides several advantages including ease of performance, lateralization of the suture line, early healing, and early return to work. Can et al. [4] reported morbidity rate of 8.9% and recurrence rate of 4.6% in their patient series, and Sakr et al. [17] reported morbidity rate of 9.7% and recurrence rate of 2.4%. In the current study, the morbidity rate was 10.5% and the recurrence rate was 2.3%. The basis for recurrence is failure to achieve efficient lateral transposition of the natal cleft at the inferior end of the incision. Moreover, extreme care should be exercised to eliminate any dead space in the wound, the wound should heal without being infected, the sacrococcygeal region should be depilated at regular intervals, and the loose hairs should be removed by daily bathing [18].

The use of surgical drains is important for preventing postoperative fluid collection. Cavity drainage is routinely used in the Karydakis method to reduce the risk of seroma formation [15]. Bessa [16] reported subcutaneous fluid collection following drainage in 2.4% of their patients. The rate of subcutaneous fluid collection was 1.55% in the current study.

Karydakis reported a hospital length of stay of 3 days [11]. In their studies, Gurer et al. [15] and Al-Jaberi [19] reported a hospital length of stay of 4 days. The mean hospital length of stay was 3.34 ± 1.42 days in the current study. The Karydakis method can also be used in the treatment of patients with recurrences previously treated by open healing or primary closure [18]. In the current study, 12 patients (4.66%) were previously treated by methods other than the Karydakis method in other centres. We utilized the Karydakis method without any difficulty in these patients.

One of the significant advantages of the Karydakis procedure is that it provides early return to daily activities in the postoperative period [2]. While this period was reported to be 3-4 weeks using the primary midline closure technique [20], it has been reported to be 12.4–20 days in the Karydakis method [21]. The time elapse to return to daily activities was 15 days in the current study. In addition, the postoperative patient satisfaction rate appears to be high by Karydakis procedure. Can et al. [4] reported the degree of satisfaction as excellent in 70.8% of patients and as good in 26.1% of patients in their study. The patient satisfaction rate was 91.06% in the current study.

The Karydakis flap procedure is a safe treatment alternative for the surgical treatment of sacrococcygeal pilonidal sinus disease because of associated low complication rate, short length of hospital stay and healing duration, and high patient satisfaction rate. 

## Figures and Tables

**Table 1:** Preoperative symptoms.

Symptoms	*n* (%)
Seropurulent discharge	148 (57.58)
Pain	108 (42.02)
Local swelling	33 (12.84)

**Table 2:** Postoperative complications.

Complications	*n* (%)
Wound infection	17 (6.61)
Seroma	4 (1.55)
Abscess	3 (1.16)

## References

[1] Petersen S, Aumann G, Kramer A, Doll D, Sailer M, Hellmich G (2007). Short-term results of Karydakis flap for pilonidal sinus disease. *Techniques in Coloproctology*.

[2] Anderson JH, Yip CO, Nagabhushan JS, Connelly SJ (2008). Day-case Karydakis flap for pilonidal sinus. *Diseases of the Colon and Rectum*.

[3] Morden P, Drongowski RA, Geiger JD, Hirschl RB, Teitelbaum DH (2005). Comparison of Karydakis versus midline excision for treatment of pilonidal sinus disease. *Pediatric Surgery International*.

[4] Can MF, Sevinc MM, Yilmaz M (2009). Comparison of Karydakis flap reconstruction versus primary midline closure in sacrococcygeal pilonidal disease: results of 200 military service members. *Surgery Today*.

[5] Kosaka M, Kida M, Mori H, Kamiishi H (2007). Pilonidal cyst of the scalp due to single minor trauma. *Dermatologic Surgery*.

[6] Can MF, Sevinc MM, Hancerliogullari O, Yilmaz M, Yagci G (2010). Multicenter prospective randomized trial comparing modified Limberg flap transposition and Karydakis flap reconstruction in patients with sacrococcygeal pilonidal disease. *American Journal of Surgery*.

[7] Keshava A, Young CJ, Rickard MJFX, Sinclair G (2007). Karydakis flap repair for sacrococcygeal pilonidal sinus disease: how important is technique?. *ANZ Journal of Surgery*.

[8] Spivak H, Brooks VL, Nussbaum M, Friedman I (1996). Treatment of chronic pilonidal disease. *Diseases of the Colon and Rectum*.

[9] Sözen S, Emir S, Güzel K, Özdemir CŞ (2011). Are postoperative drains necessary with the Karydakis flap for treatment of pilonidal sinus? (Can fibrin glue be replaced to drains?) A prospective randomized trial. *Irish Journal of Medical Science*.

[10] Karydakis GE (1973). New approach to the problem of pilonidal sinus. *The Lancet*.

[11] Karydakis GE (1992). Easy and successful treatment of pilonidal sinus after explanation of its causative process. *Australian and New Zealand Journal of Surgery*.

[12] Hull TL, Wu J (2002). Pilonidal disease. *Surgical Clinics of North America*.

[13] Kitchen PRB (1996). Pilonidal sinus: experience with the Karydakis flap. *British Journal of Surgery*.

[14] Tezel E, Bostanci H, Azili C, Kurukahvecioğlu O, Anadol Z (2009). A new perspective for pilonidal sinus disease and its treatment. *Marmara Medical Journal*.

[15] Gurer A, Gomceli I, Ozdogan M, Ozlem N, Sozen S, Aydin R (2005). Is routine cavity drainage necessary in Karydakis flap operation? A prospective, randomized trial. *Diseases of the Colon and Rectum*.

[16] Bessa SS (2007). Results of the lateral advancing flap operation (modified Karydakis procedure) for the management of pilonidal sinus disease. *Diseases of the Colon and Rectum*.

[17] Sakr M, El-Hammadi H, Mousa M, Arafa S, Rasheed M (2003). The effect of obesity on the results of Karydakis technique for the management of chronic pilondal sinus. *International Journal of Colorectal Disease*.

[18] Akinci OF, Bozer M, Uzunköy A, Düzgün SA, Coşkun A (1999). Incidence and aetiological factors in pilonidal sinus among Turkish soldiers. *European Journal of Surgery*.

[19] Al-Jaberi TMR (2001). Excision and simple primary closure of chronic pilonidal sinus. *European Journal of Surgery*.

[20] Gencosmanoglu R, Inceoglu R (2005). Modified lay-open (incision, curettage, partial lateral wall excision and marsupialization) versus total excision with primary closure in the treatment of chronic sacrococcygeal pilonidal sinus: a prospective, randomized clinical trial with a complete two year follow-up. *International Journal of Colorectal Disease*.

[21] Akinci OF, Coskun A, Uzunköy A (2000). Simple and effective surgical treatment of pilonidal sinus: asymmetric excision and primary closure using suction drain and subcuticular skin closure. *Diseases of the Colon and Rectum*.

